# *Pseudomonas aeruginosa* Ventilator-Associated Pneumonia Induces Lung Injury through TNF-α/c-Jun NH2-Terminal Kinase Pathways

**DOI:** 10.1371/journal.pone.0169267

**Published:** 2017-01-06

**Authors:** Ying-Wei Yang, Yu-Zhen Jiang, Ching-Mei Hsu, Lee-Wei Chen

**Affiliations:** 1 Institute of Emergency and Critical Care Medicine, National Yang-Ming University, Taipei, Taiwan; 2 Department of Anesthesiology, Mackay Memorial Hospital, Taipei, Taiwan; 3 Department of Biological Sciences, National Sun Yat-Sen University, Kaohsiung, Taiwan; 4 Department of Surgery, Kaohsiung Veterans General Hospital, Kaohsiung, Taiwan; Hospital for Sick Children, CANADA

## Abstract

Ventilator-associated pneumonia (VAP) is a common nosocomial infection among intensive care unit (ICU) patients. *Pseudomonas aeruginosa* (PA) is the most common multidrug-resistant Gram-negative pathogen and VAP caused by PA carries a high rate of morbidity and mortality. This study examined the molecular mechanism of PA VAP-induced lung injury. C57BL/6 wild-type (WT) mice and JNK1 knockout (JNK1^-/-^) mice received mechanical ventilation (MV) for 3 h at 2 days after receiving nasal instillation of PA. The WT and JNK1^-/-^ mice also received MV after the induction of lung injury by instillation of supernatants from PA-stimulated alveolar macrophages (AMs). AMs isolated from WT, IκB-kinase (IKK)β^ΔMye^ (IKKβ was selectively deleted in macrophages), and JNK1^-/-^ mice were *ex vivo* stimulated with live PA and supernatants were collected for cytokine assay. Intranasal instillation of 10^6^ PA enhanced MV-induced NF-κB DNA binding activity in the lungs and nitrite levels in BALF. MV after PA instillation significantly increased the expression of ICAM and VCAM in the lungs and TNF-α, IL-1β, and IL-6 levels in bronchoalveolar lavage fluid (BALF) of WT mice, but not in JNK1^-/-^ mice. MV after supernatant instillation induced more total protein concentration in BALF and neutrophil sequestration in the lungs in WT mice than JNK1^-/-^ mice and cytokine assay of supernatants indicated that TNF-α is a critical regulator of PA VAP-induced lung injury. E*x vivo* PA stimulation induced TNF-α production by AMs from WT as well as JNK1^-/-^ mice but not IKKβ^ΔMye^ mice. In summary, PA colonization plays an important role in PA VAP-induced lung injury through the induction of JNK1-mediated inflammation. These results suggest that the pathogenesis mechanism of PA VAP involves production of TNF-α through activation of IKK/NF-κB pathways in AMs and JNK signaling pathway in the lungs.

## Introduction

Ventilator-associated pneumonia (VAP) is a serious complication in patients receiving MV longer than 48 h [1]. PA is responsible for a high proportion of these infections in hospitalized patients [2]. The tracheobronchial colonization is one of the most important factors for VAP and the predominant organisms responsible for infection are *Staphylococcus aureus*, PA and *Enterobacteriaceae*. Large quantities of PA in the trachea of ventilated patients are associated with an increased risk of death [3]. A recent study has reported that higher percentages of *Pseudomonas aeruginosa* colonized patients subsequently developed *Pseudomonas aeruginosa* VAP [4]. Thus, the role of PA colonization in PA VAP-induced lung injury remains to be clearly defined.

Studies in human subjects indicate that the release of cytokines/chemokines and the recruitment of leukocytes causes ventilator-associated lung injury (VALI) [5]. Experimental models have demonstrated increased vascular permeability, higher cell count and protein concentration in the bronchoalveolar lavage fluid (BALF), and increased inflammatory cell infiltration into lung tissues in ventilator-induced lung injury (VILI) [5–9]. Experimental models of ventilator-induced lung injury (VILI) were characterized with increased vascular permeability, higher cell count and protein concentration in the bronchoalveolar lavage fluid (BALF), and increased inflammatory cell infiltration into lung tissues [5–9]. A lot of diseases such as autoimmune diseases, cancer and inflammatory diseases are associated with alterations in NF-κB activity [10]. Furthermore, a few experimental models have demonstrated that blockage of NF-κB activation decreases the VILI [5]. Activation of NF-κB dimers results from IκB-kinase (IKK)-mediated phosphorylation-induced proteasomal degradation of IκB, allowing the active NF-κB transcription factor subunits to translocate to the nucleus and induce target gene expression [11]. Studies have demonstrated that macrophage inflammatory protein (MIP-2), interleukin-6 (IL-6), and tumor necrosis factor-α (TNF-α) are involved in inflammation [12,13]. MIP-2 is the most potent leukocyte chemoattractant and plays a very important role in the pathogenesis of VILI [5]. Kinases of the JNK group are primarily activated by stress stimuli (UV radiation, pH changes, heat shock, as well as genotoxic and oxidative stress) and proinflammatory cytokines (TNF-α and IL-1) in mammalian cells [14]. It has been reported that JNKs are specifically relevant to TNF-α-mediated induction of AP-1 activity [15,16]. However, the different roles of NF-κB and JNK in VILI have not been well characterized.

Airway epithelial cells are the front line defender of the lungs to against invading microbes by providing the physical barrier and antimicrobial activity [17]. The airway epithelial cells increase production of mediators such as cytokines, chemokines and antimicrobial peptides to respond to such exposure [18]. In response to pathogens, the endothelial cells promote inflammation by expressing different combinations of adhesion molecules for leukocytes such as E-selectin, intercellular adhesion molecule-1 (ICAM-1) and vascular cell adhesion molecule-1 (VCAM-1) in distinct temporal, spatial and anatomical patterns [19]. AMs play a role in regulation of innate immunity in respiratory system by synthesizing and secreting pro- and anti-inflammation cytokines/chemokines [20]. AMs represent the first line of lung defense against pathogens such as PA and epithelial cells promote neutrophil sequestration into lungs which may cause lung injury and active the host immune response. The role of AMs in PA VAP-induced lung injury remains uncertain. Therefore, in this study, the nasal instillation of PA, TNF-α protein, or supernatants from *ex vivo* PA-stimulated AMs before MV in mice was used as models to study the mechanism of PA VAP-induced lung injury. The primary objective of this study was to determine the relationship between PA colonization and VAP-induced lung injury. The secondary objective was to examine the molecular mechanisms and involvement of TNF-α as well as JNK in PA VAP-induced lung injury. We found that PA VAP-induced lung injury involved the TNF-α production from AMs and JNK signaling pathway in the lungs. Using JNK inhibitor in ICU patients with higher percentages of PA colonization may reduce VAP-induced lung injury and mortality.

## Materials and Methods

### Mice

C57BL/6 (wild-type, WT) mice (total n = 360) weighing between 18 g and 25 g were purchased from the National Laboratory Breeding and Research Center (NLBRC, Taipei, Taiwan). IKKβ^ΔMye^ (Cre-*lox* mediated gene targeting, IKKβ was selectively deleted in macrophages) mice (total n = 36) and JNK1^-/-^ (c-Jun N-terminal kinases knockout) mice (total n = 180) generated from the same background were transferred from Dr. Karin’s laboratory (University of California, San Diego, CA, USA). All animal procedures were in compliance with the regulations on animals used for experimental and other scientific purposes approved by the Kaohsiung Veterans General Hospital Animal Experiments Committee.

### Ethics statement

This study was approved by the Institutional Animal Care and Use Committee of Kaohsiung Veterans General Hospital (Permit Number: VGHKS-104-A018), and animal experiments were performed according to Animal Experimentation Regulations of Kaohsiung Veterans General Hospital. All efforts were made to minimize suffering.

In the survival experiments, animals were checked every 6 hours for signs of distress and endpoints. Specific criteria used to determine when the animals should be euthanized were in accordance with Remick lab report [21]. Mice were systematically euthanized with avertin when they were found in a moribund state as identified by inability to maintain upright associated or not with labored breathing and cyanosis. Classical signs of distress such as anorexia and weight loss (> 20%), hunching, prostration, impaired motility, labored breathing, ruffled haircoat, dehydration, were assessed. Mice exhibiting at least four of these criteria were humanely euthanized with avertin and cervical dislocation. Euthanized mice were considered as non-survivors.

### PA VAP-induced lung injury

**Fig 1:**
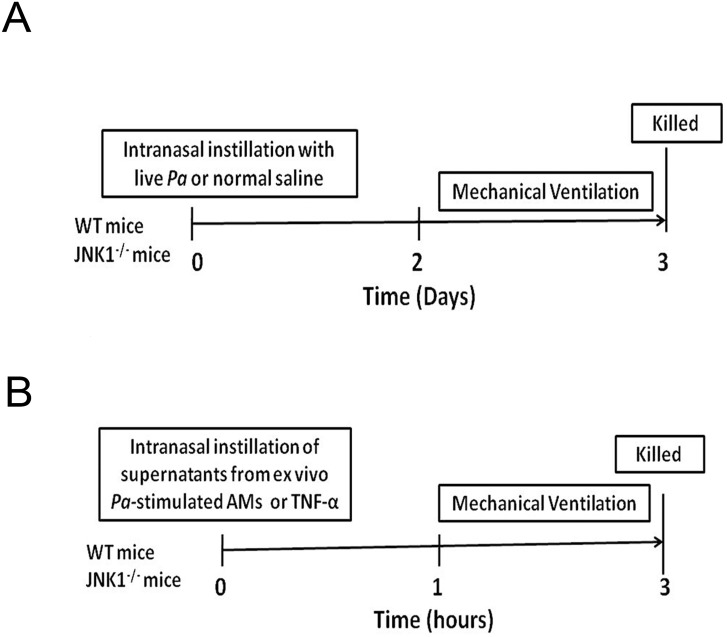
Animal models in examining PA VAP-induced lung injury. (A) Animals were instilled with 10 μl of normal saline as the control or with equal volume of PA (ATCC 27853, 10^4^ or 10^6^ CFU) via nostrils into the lungs. Two days after PA instillation, mice received MV with high tidal volume for 3 hr. (B) To examine the role of AMs on PA VAP-induced lung injury, AMs collected from BALF of WT mice were stimulated with live PA for 4 h. The anesthetized mice were instilled with 10 μl of supernatants and received MV for 3 hr at 1 hr after supernatant instillation. PA, *P*. *aeruginosa*; VAP, Ventilator-associated pneumonia; AMs, alveolar macrophages; BALF, bronchoalveolar lavage fluid; NS, normal saline.

An animal model of PA VAP-induced lung injury was established. WT mice were anesthetized with avertin (15 mg/kg, Sigma) and instilled with 10 μl of normal saline as the control or with equal volume of PA (ATCC 27853, 10^4^ or 10^6^ CFU) via nostrils into the lungs. Two days after PA instillation, mice received MV with high tidal volume for 3 hr. Mice were sacrificed and the lungs were harvested and assayed for neutrophil infiltration, proinflammatory cytokines/chemokines expression, NF-κB and AP-1 DNA-binding activity, and histological study (Fig 1A). BALF was also collected for cell counting and nitrite as well as cytokines assay.

### MV treatment

At 2 days after instillation, mice were sacrificed or received ventilation for 3 hr. Mice were anaesthetized with avertin (15 mg/kg, Sigma) and the neck was cut at 1 cm below the mouth. Muscles were separated and the trachea was opened and cannulated with a 0.5 cm 21G needle which is connected to a mechanical ventilator (SAR-830/P, CWE Inc., Ardmore, PA, USA) for 3 hr. During the ventilation period, mice were monitored every 30 mins. The ventilation was set at a respiration rate of 160 breaths/minute for 3 h with zero end expiratory pressure. The tidal volume was 21 ml/kg.

### MV after instillation of supernatants from ex vivo PA–stimulated AMs

To examine the role of AMs on PA VAP-induced lung injury, AMs collected from BALF of WT mice were stimulated with live PA for 4 h. After stimulation, supernatants were collected by centrifugation at 9,500 × g for 15 min and assayed for cytokines by enzyme-linked immunosorbent assay (ELISA) and nitrite by Griess reagent. The anesthetized WT mice were instilled with 10 μl of supernatants and received MV for 3 hr at 1 hr after supernatant instillation (Fig 1B).

### Tissue preparation

Mice were sacrificed and the lungs and heart were harvested. Saline (5 ml) was injected into right ventricle by syringe to clear of the blood in pulmonary vasculature. The lung tissue was blotted dry of surface blood and immediately stored at -80°C for analysis.

### Assay for bacterial counts in the lungs

Lung tissues were weighed and homogenized in saline. The homogenized lung samples were diluted and plated (100 μl) onto LB agar plates. The plates were examined for bacterial growth after overnight aerobic incubation at 37°C. Data were expressed as the numbers of colony forming unit per gram of lung tissue (CFU/g tissue).

### Neutrophil infiltration in the lungs

Pulmonary myeloperoxidase (MPO) activity is a marker of lung neutrophil infiltration [22]. Lung tissues were weighed and homogenized in 50 mM potassium phosphate buffer (pH 6.0) with 0.5% hexadecyltrimethyl- ammonium bromide. Homogenates were centrifuged at 9,500 × g, 4°C for 10 min. An aliquot of supernatants (60 μl) was added to 939 μl of potassium phosphate buffer with 16.7 mg/ml of O-dianisidine and 0.5% hydrogen peroxide. The rate of change in absorbance at 460 nm was measured over 2 min. One unit of MPO activity is defined as the amount of enzyme that reduces 1 μmole of peroxide per min and the data were expressed as units per gram of lung tissue (Units/g tissue).

### Preparation of BALF

For whole lung lavage, the lavage was washed with two separate injections of 0.5 ml sterile saline through a 21G needle that was cannulated 0.5 cm into the trachea. The collected BALF was used for cell counting with a hemocytometer. BALF was also centrifuged at 350 × g for 5 min. The supernatants were collected and stored at -80°C for cytokine analysis by ELISA or for nitrite production assay by using Griess reagent. The pellet was used for *ex vivo* stimulation assay of AMs.

### Ex vivo stimulation of AMs with PA

Pellets collected after centrifugation of BALF were suspended with RPMI 1640 (Sigma) in 96-well microtiter plates (200 μl/well) and cultured for 2 hr at 37°C for attachment. Nonattached cells were washed away and the attached cells (AMs) were stimulated with or without live PA (10^4^ or 10^6^ CFU in 200 μl) for 4 hr. After stimulation, the supernatants were collected, incubated at 65°C in a water bath for 1 h, and centrifuged at 9,500 × g for 15 min to remove PA. The samples were used for instillation in mice or stored at -80°C for ELISA and Griess reagent assay. For instillation in mice, 10 μl of the supernatants was instilled into the lungs via nostrils at 1 hr before ventilation.

### Reverse Transcription Polymerase Chain Reaction (RT-PCR)

The total RNAs were isolated from lung tissues using total RNA Miniprep Purification Kit (GeneMark). The cDNAs encoding proinflammatory cytokines and chemokines were generated by reverse transcription and amplified by PCR. Sets of IL-6, ICAM, VCAM, MIP-2, and GADPH (Glyceraldehyde-3-phosphate dehydrogenase) primers were designed according to those genes documented in the GenBank.

For the PCR reaction, to the 0.2 ml tubes were added 3 μl of 10× Gene Taq buffer (GeneMark Inc. Atlanta, USA), 2 μl of 2.5 mM dNTP, 0.5 μl of 25 mM sense and anti-sense primers and an appropriate amount of water to make a total volume of 30 μl. After adding 0.05 μl of Gene Taq DNA polymerase (5 U/μl), amplification was performed in a thermocycler (Bio-Rad) with the following profile: 5 min at 95°C before the first cycle, 1 min at 95°C for denaturation, 1 min at 58°C for annealing, and 1 min 30 second at 72°C for extension, finally 10 min at 72°C after the last cycle.

### Enzyme-Linked Immunosorbent Assay (ELISA)

BALF and supernatants collected after *ex vivo* stimulation of AMs were used for TNF-α, IL-1β and IL-6 assay by using the mouse ELISA kit (eBioscience). Lung tissue was homogenized in lysis buffer (30 mM Tris, pH 7.5, 300 mM NaCl, 2 mM MgCl_2_, 10% Triton X-100, 2 mM CaCl_2_, and 20 μg/ml of protease inhibitors) and centrifuged at 1,000 × g, 4°C for 15 min. The supernatants was collected and used for assay. The ELISA plates were coated with capture antibodies (100 μl per well) at 4°C for overnight. The plates were washed several times and blocked with assay buffer (200 μl per well) at room temperature for 1 hr. The samples and standards were added to the plates and incubated at 4°C for overnight. On the next day, the plates were washed several times, detection antibodies (100 μl per well) were added for 1 hr and avidin-HRP (100 μl per well) was added for 30 min at room temperature. Finally, substrate 3,3',5,5'-tetramethylbenzidine was added and incubated at room temperature for 15 min. The reaction was stopped by adding 2 N H_2_SO_4_ and the absorbance at 450 nm was measured by using an ELISA reader.

### Electrophoretic Mobility Shift Assay (EMSA) for NF-κB and AP-1

Tissue nuclear extract was obtained by using NE-PER nuclear and cytoplasmic extraction reagents (CER I, CER II and NER, Pierce). Tissue was added 150 μl of CER I containing protease inhibitors and homogenized. Sample was vortexed vigorously for 15 sec to fully resuspend the tissue and incubated on ice for 10 min. The mixture was added 11 μl of CER II, vortexed for 5 sec, and incubated on ice for 1 min, then vortexed for 5 sec, and finally centrifuged at 9,500 × g, 4°C for 7 min. The supernatants (cytoplasmic extract) were stored and the pellet containing nuclei was resuspended in 50 μl of NER. The suspension was centrifuged at 9,500 × g, 4°C for 12 min and the supernatant fraction (nuclear extract) was immediately transferred to a clean tube and stored at -80°C until use.

The double-stranded DNA probe containing the NF-κB binding consensus sequence (5’CGCTTCATGACTTGGCCGGAACGCTTG-ATGACTTGGCCGGAA3’) or AP-1 binding consensus sequence (5’CGCTTCATGACTTGGCCGGAACGCTTG-ATGACTTGGCCGGAA3’) were end-labeled with 5 μM biotin-11-UTP (Pierce) at 37°C for 30 min by using terminal deoxynucleotidyl transferase (2 U/μl) and the labeling was stopped by adding 2.5 μl of 0.2 M EDTA.

The biotin-labeled probe was incubated with 5 μg of tissue nuclear protein for binding at room temperature for 20 min. The reaction mixtures were electrophoresed at 100 V on a 4% nondenaturing polyacrylamide gel. The samples were transferred onto the nylon membrane at 380 mA for 1 hr. The membrane was placed to face down on a transilluminator which equipped with 312 nm bulbs and be crosslinked for 15 min. The membrane was washed few times and removed to a new container. The substrate equilibration buffer was added for 5 min with gentle shaking. The cemiluminescent substrate working solution was added and the membrane was placed in a film cassette and exposed to X-ray film for detection.

### Statistics

All data are analyzed by one-way analysis of variance or T-test analysis of variance (ANOVA), followed by Turkey’s Multiple Comparison Test. All values in the figures and text are expressed as mean ± standard error of the mean. P values of less than 0.05 are considered to be statistically significant.

## Results

### MV after PA instillation induces neutrophil infiltration in the lungs and nitrite in BALF

**Fig 2:**
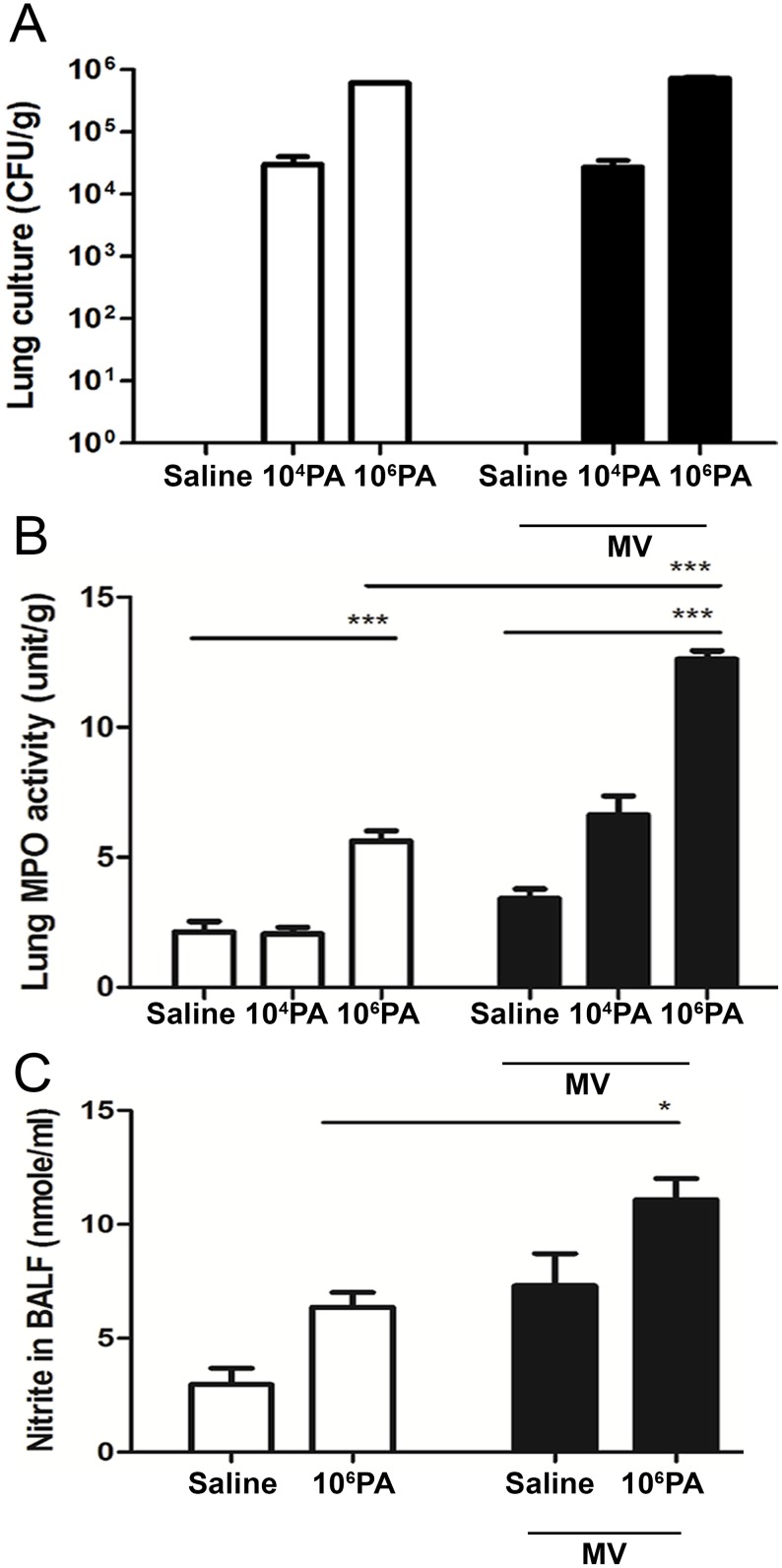
Effects of *P*. *aeruginosa* (PA) instillation on mechanical ventilation-induced lung injury. (A) WT mice were intranasally instilled with live PA (1 × 10^4^ or 1 × 10^6^ CFU) or normal saline at 2 days before receiving mechanical ventilation (MV) for 3 h and lung tissues were harvested and assayed. (B) Positive bacterial cultures of lung tissues shown as CFU counted on the LB plates. (C) PA instillation before ventilation increased MPO activity of the lung. (D) PA instillation before MV increased nitrite concentration in BALF. Nitrite in BALF was assayed by Griess reagent. PA, *P*. *aeruginosa*; MPO, myeloperoxidase; BALF, bronchoalveolar lavage fluid; NS, normal saline. *P<0.05, **P<0.01, ***P<0.001. n = 6/group.

Normal saline intranasal instillation did not induce PA CFUs in the lungs. Instillation with 10^4^ or 10^6^ PA induced PA CFUs in the lungs (Fig 2A). Mechanical ventilation for 3 h at 2 days after 10^4^ or 10^6^ PA instillation did not increase PA CFUs in the lungs when compared with mice that did not receive mechanical ventilation. This observation indicates that mechanical ventilation after PA instillation did not induce PA overgrowth in the lungs. The MPO lung activity shows no difference between control and 10^4^ PA instillation group but significant increase in 10^6^ PA instillation group. This result indicates that PA-induced neutrophil recruitment in the lung is very sensitive for the bacterial counts in the lung. MV after PA instillation induced a significant increase of lung MPO activity as compared with PA instillation or MV alone group (Fig 2B). MV after normal saline instillation did not significantly induce MPO activity in the lungs as compared with the saline instillation group. These results indicate that MV after PA instillation enhances PA-induced neutrophil infiltration in the lungs. Moreover, MV after PA instillation induces neutrophil infiltration in the lungs through mechanisms other than enhancing PA overgrowth and PA instillation enhances MV-induced lung injury.

The nitrite concentration in BALF was also significantly increased (1.5 to 2-fold) in mice receiving MV after 10^6^ PA instillation as compared with mice receiving PA instillation alone (Fig 2C). These results suggest that MV enhances PA instillation-induced nitrite production in BALF.

### MV after PA instillation induces ICAM and VCAM expression in the lungs

**Fig 3:**
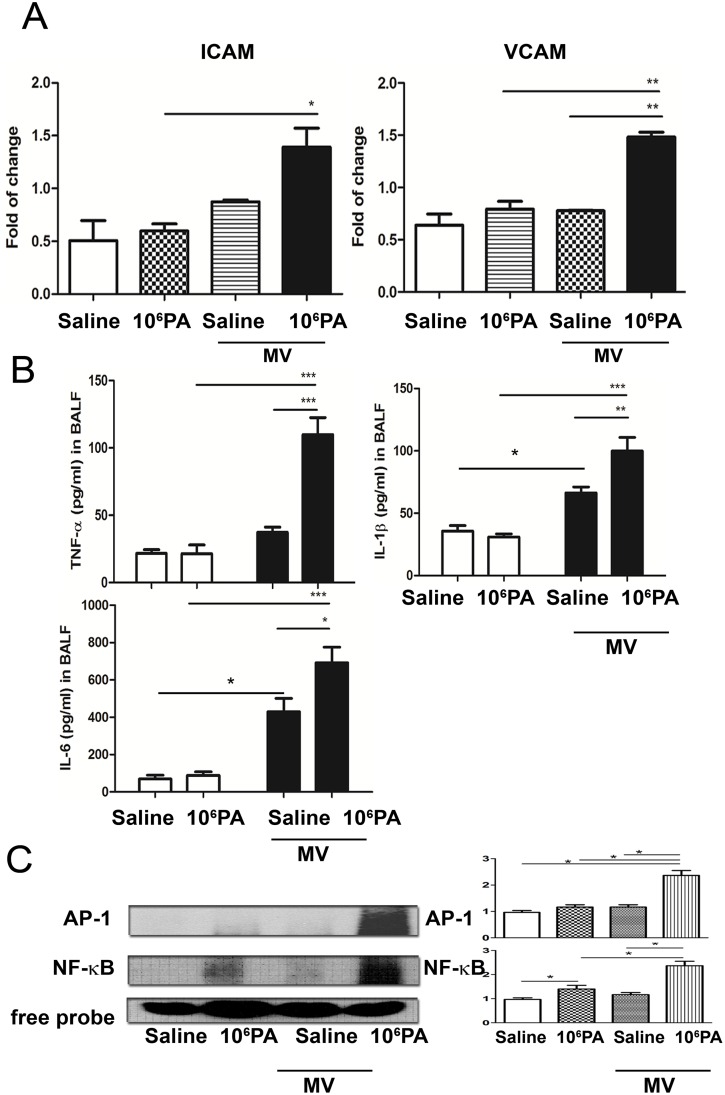
Mechanical ventilation (MV) after PA instillation induced mRNA expression of cytokines and NF-κB as well as AP-1 DNA-binding activity in the lungs. (A) MV after PA instillation significantly induced mRNA expression of ICAM and VCAM in the lungs in WT mice as compared with PA instillation or MV alone group. n = 5/group. (B). MV after PA instillation induced TNF-α, IL-1β and IL-6 production in BALF of WT mice as compared to those in PA instillation or MV alone group. The concentrations of TNF-α, IL-1β and IL-6 in the BALF were determined by enzyme-linked immunosorbent assay (ELISA). n = 5/group. (C) The DNA-binding activity of NF-κB and AP-1 in the lungs of WT mice receiving MV after normal saline or PA (10^4^ or 10^6^ CFU) instillation. Signal intensity was determined by densitometry and normalized to free probe band. *P<0.05, **P<0.01. n = 4/group. CFU, colony forming unit; NS, normal saline. *P<0.05, **P<0.01.

To study the role of proinflammatory chemokines in PA VAP-induced lung injury, ICAM and VCAM mRNA in the lungs were analyzed. MV or PA instillation did not induce ICAM and VCAM mRNA expression in the lungs. The expression of ICAM and VCAM mRNA in the lungs was significantly increased in mice receiving MV after PA (10^6^ CFU) instillation as compared with those receiving PA instillation alone (Fig 3A). These results indicate that PA instillation enhances MV-induced ICAM and VCAM mRNA expression in the lungs.

### MV after PA instillation induces cytokine production in the BALF

To study the role of cytokines in PA VAP-induced lung injury, the concentrations of TNF-α, IL-1β and IL-6 in the BALF were determined by enzyme-linked immunosorbent assay (ELISA). MV induced a significant increase of IL-6 and IL-1β levels in BALF as compared with sham group (Fig 3B). MV after PA instillation in mice induced a significant increase of TNF-α, IL-1β, and IL-6 levels in the BALF as compared with those receiving ventilation or PA instillation alone (Fig 2B). These results indicate that MV induces IL-6 and IL-1β levels in BALF and PA instillation enhances MV-induced TNF-α, IL-1β and IL-6 levels.

### MV after PA instillation induces NF-κB and AP-1 DNA binding activity in the lungs

NF-κB and AP-1 are key transcription factors that regulate the expression of several genes involved in inflammation [23]. PA instillation induced an increase of NF-κB DNA binding activity in the lungs as compared with saline instillation group (Fig 3C). MV alone did not induce NF-κB DNA binding activity in the lungs. Mechanical ventilation or PA nasal instillation did not induce AP-1 DNA-binding activity in the lungs. However, mechanical ventilation after PA instillation significantly induced NF-κB and AP-1 DNA-binding activity in the lungs as compared with mice receiving MV or PA instillation alone (Fig 3C). These results indicate that PA instillation enhances MV-induced NF-κB and AP-1 DNA binding activity in the lungs.

### PA stimulation of AMs induced TNF-α and nitrite levels in the supernatants

**Fig 4:**
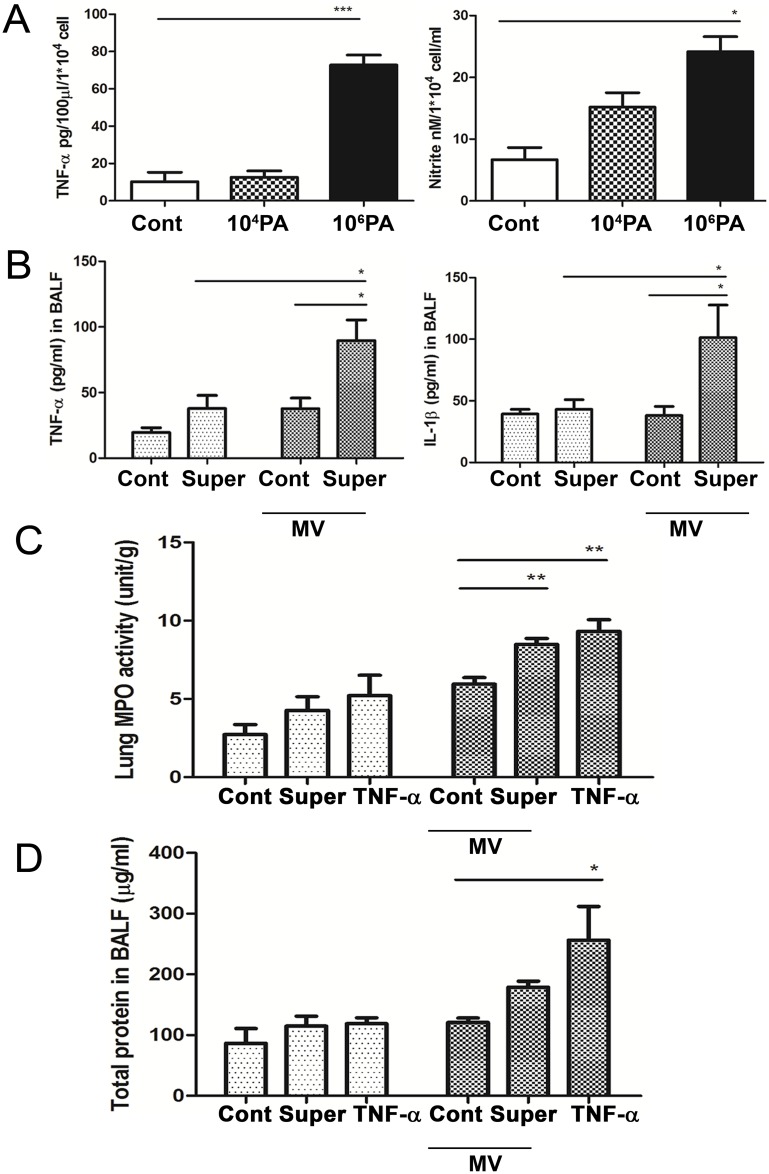
Instillation of supernatants from *ex vivo* PA-stimulated AMs enhanced mechanical ventilation-induced lung injury. (A) AMs collected from BALF of WT mice were stimulated with live PA for 4 h. After stimulation, supernatants were collected for TNF-α analysis by ELISA or for nitrite production assay by using Griess reagent. n = 6/group. (B) Mechanical ventilation (MV) after instillation of supernatants from *ex vivo* PA-stimulated AMs increased TNF-α as well as IL-1β levels in BALF. The concentrations of TNF-α and IL-1β in the BALF were determined by enzyme-linked immunosorbent assay (ELISA). Mice were instilled with supernatants from PA-stimulated AMs and received MV for 3 h at 1 h after supernatant instillation. n = 4/group. (C) MV after instillation of TNF-α (100 ng) or supernatants from *ex vivo* PA-stimulated AMs induced pulmonary MPO activity. Mice were instilled with TNF-α or supernatants from PA-stimulated AMs and received MV for 3 h at 1 h after supernatant instillation. n = 6/group. (D) MV after instillation of supernatants from *ex vivo* PA-stimulated AMs or TNF-α (100 ng) induced total protein concentration in BALF. n = 5/group. PA, *P*. *aeruginosa*; Super, supernatants from *ex vivo* PA stimulation of AMs. *P<0.05, **P<0.01, ***P<0.001.

To determine which proinflammatory cytokines play major roles in MV after PA colonization-induced lung injury, AMs of WT mice were *ex vivo* stimulated with PA (10^4^ or 10^6^ CFU) and the supernatants were examined for TNF-α, IL-1β, IL-6 and nitrite levels. Stimulation of AMs with 10^6^ PA significantly induced TNF-α and nitrite levels in the supernatants (Fig 4A) but had no effect on the production of IL-1β and IL-6 levels (not shown). These results indicate that ex vivo PA stimulation of AMs induces a significant increase of TNF-α and nitrite levels in the supernatants.

### MV after instillation of supernatants from ex vivo PA-stimulated AMs induces TNF-α and IL-1β production in the lungs

To further examine the involvement of AMs in PA VAP-induced lung injury, mice were instilled with supernatants from PA-stimulated AMs and received MV for 3 h at 1 h after supernatant instillation. MV after supernatant instillation induced a significant increase in the TNF-α and IL-1β levels in the BALF as compared with those receiving supernatant infiltration or MV alone (Fig 4B). These results indicate that supernatants from ex vivo PA-stimulated AMs induce similar effects as PA instillation and mediators from PA-stimulated AM enhance MV-induced lung inflammation.

### MV after instillation of TNFα or supernatant induces neutrophil infiltration and protein concentrations in the lungs

To further examine the involvement of TNF-α in PA VAP-induced lung injury, mice were instilled with TNF-α and received MV for 3 h at 1 h after TNF-α instillation. MV after instillation of supernatants from ex vivo PA-stimulated AMs induced a significant increase of MPO activity as compared with those receiving vehicle instillation (Fig 4C). Therefore, TNF-α protein (100 ng in 10μl) was used to replace the supernatants for instillation to examine the role of TNF-α in MV after PA instillation-induced lung injury. Similar with PA or supernatant instillation, TNF-α instillation significantly enhanced MV-induced MPO activity (Fig 4C) in the lungs as well as total protein concentration (Fig 4D) in the BALF as compared with those receiving ventilation alone (Fig 4C & 4D), and the increases were higher in mice receiving TNF-α instillation. These results suggest that instillation of TNF-α or supernatants from ex vivo PA-stimulated AMs enhance MV-induced lung injury.

### MV after PA instillation induces cytokine expression in the lungs of WT mice but not in JNK1^-/-^ mice

**Fig 5:**
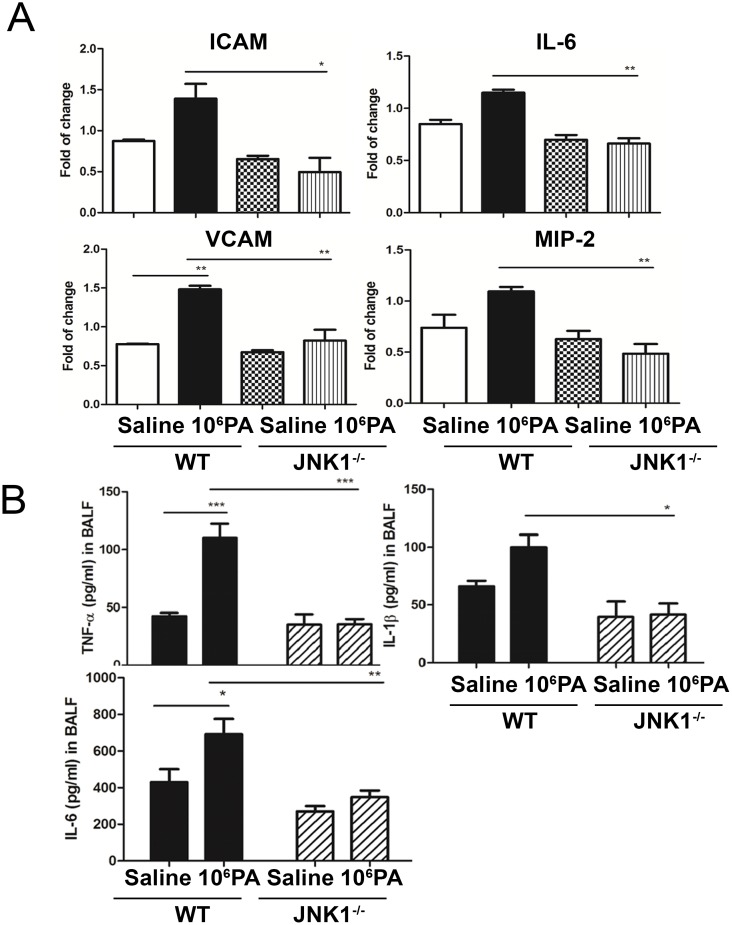
Mechanical ventilation (MV) after PA instillation induced mRNA expression of cytokines in the lungs in WT mice but not in JNK1^-/-^ mice. (A) mRNA expression of ICAM, VCAM, IL-6, and MIP-2 in the lungs in WT mice and JNK1^-/-^ mice after PA instillation and MV treatment. Signal intensity was determined by densitometry and normalized to GAPDH band. n = 4/group. (B) The levels of proinflammatory cytokines (TNF-α, IL-1β and IL-6) in BALF in WT and JNK1^-/-^ mice after PA instillation and MV treatment. The concentrations of TNF-α, IL-1β and IL-6 in the BALF were determined by enzyme-linked immunosorbent assay (ELISA). n = 5-6/group. *P<0.05, **P<0.01.

To examine the role of JNK signaling pathway in PA VAP-induced lung injury, the expression of cytokines in the lungs were determined. The expression of IL-6, ICAM, VCAM and MIP-2 mRNA in the lungs of MV with PA instillation group in JNK1^-/-^ mice was significantly decreased as compared with WT mice (Fig 5A). The concentrations of TNF-α, IL-1β and IL-6 in BALF of MV with PA instillation group in JNK1^-/-^ mice were also significantly decreased as compared with WT mice (Fig 5B). These results suggest that JNK signaling pathway is crucial in MV after PA instillation-induced production of proinflammatory cytokine/chemokine in the lungs and BALF.

### Supernatants from ex vivo PA-stimulated AMs induces TNF-α levels and total protein concentration in BALF of WT mice but not in JNK1^-/-^ mice

**Fig 6:**
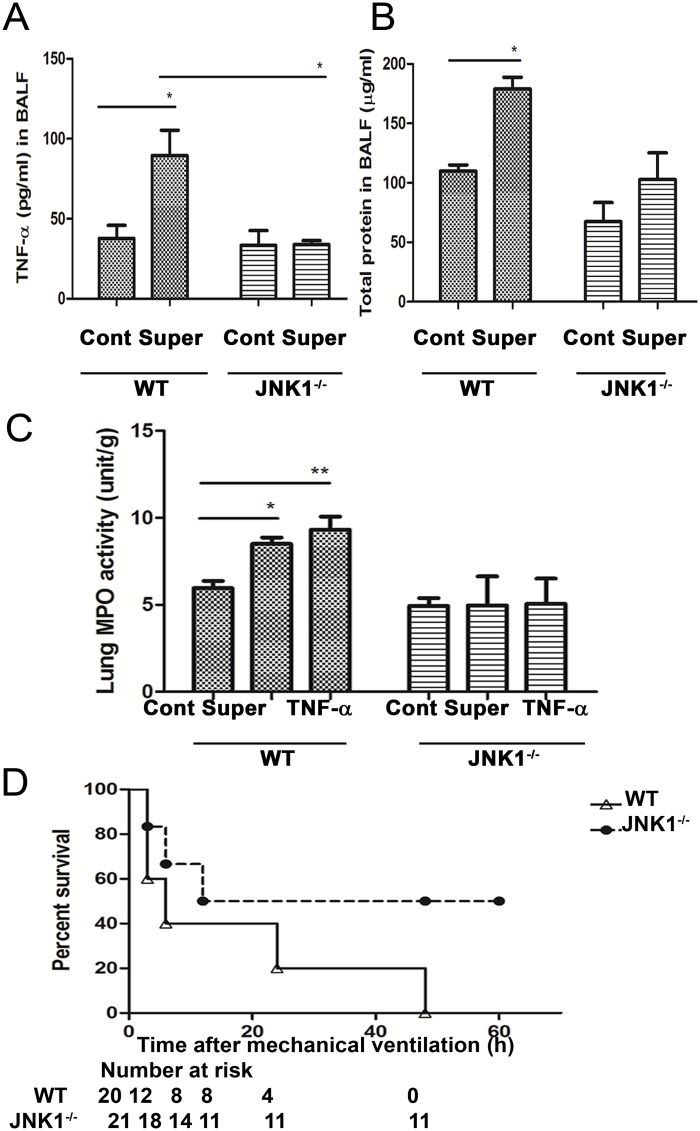
Effects of mechanical ventilation (MV) after instillation of supernatants from *ex vivo* PA stimulated AMs on pulmonary MPO activity and total protein concentration in BALF in WT and in JNK1^-/-^ mice. MV after supernatant instillation induced TNF-α levels (A) as well as total protein concentration (B) in BALF in WT mice but not in JNK1^-/-^ mice. n = 6/group. The concentrations of TNF-α in the BALF were determined by enzyme-linked immunosorbent assay (ELISA). (C) MV after supernatant instillation induced pulmonary MPO activity in WT mice but not in JNK1^-/-^ mice. Also, MV after TNF-α instillation induced lung MPO activity in WT mice but not in JNK1^-/-^ mice. Mice were instilled with TNF-α or supernatants from PA-stimulated AMs and received MV for 3 h at 1 h after supernatant instillation. n = 5-6/group. (D) Mechanical ventilation after instillation of supernatants from *ex vivo* PA stimulation of AMs induced a higher mortality in WT mice than in JNK1^-/-^ mice. PA, *P*. *aeruginosa*; Super, supernatants from *ex vivo* PA stimulation of AMs. *P<0.05, **P<0.01, ***P<0.001.

To examine the role of JNK signaling pathway in MV after supernatant instillation-induced lung injury, TNF-α levels and total protein concentration in BALF of JNK1^-/-^ and WT mice were examined. MV after supernatant instillation induced a significant increase of TNF-α levels (Fig 6A) and total protein concentration (Fig 6B) in BALF as compared with those receiving MV after vehicle instillation of WT mice but not in JNK1^-/-^ mice. This indicates that the JNK signaling pathway in the lungs plays an important role in MV with supernatant instillation-induced TNF-α levels and total protein concentration in the BALF.

### MV after instillation of TNF-α or supernatants from ex vivo PA-stimulated AMs induces MPO activity of WT mice but not in JNK1^-/-^ mice

MV after instillation of supernatants from *ex vivo* PA-stimulated AMs induced a significant increase of pulmonary MPO activity as compared with those receiving MV after vehicle instillation of WT mice but not in JNK1^-/-^ mice (Fig 6C). Similarly, MV after TNF-α instillation induced a significant increase of pulmonary MPO activity of WT mice but not in JNK1^-/-^ mice (Fig 5C). These results indicate that JNK signaling pathway in the lungs is important in MV after supernatants or TNF-α instillation-induced neutrophil infiltration.

### Mechanical ventilation after supernatant instillation induces less mortality in JNK1^−/−^ mice than in WT mice

The survival rates of WT and JNK1^−/−^ mice receiving mechanical ventilation after instillation of supernatants from *ex vivo* PA-stimulated AMs were examined. Mechanical ventilation after supernatant instillation induced a 100% mortality rate at 48 hr after ventilation in the WT mice. However, JNK1^−/−^ mice had only 50% mortality rate (Fig 6D). These animals died of respiratory failure. This observation indicates that mechanical ventilation after supernatant instillation induces less mortality rate in JNK1^−/−^ mice than WT mice.

### Ex vivo PA stimulation of AMs from IKKβ^ΔMye^ mice induces less TNF-α production in supernatants than those from IKKβ^ΔMye +/-^ mice

**Fig 7:**
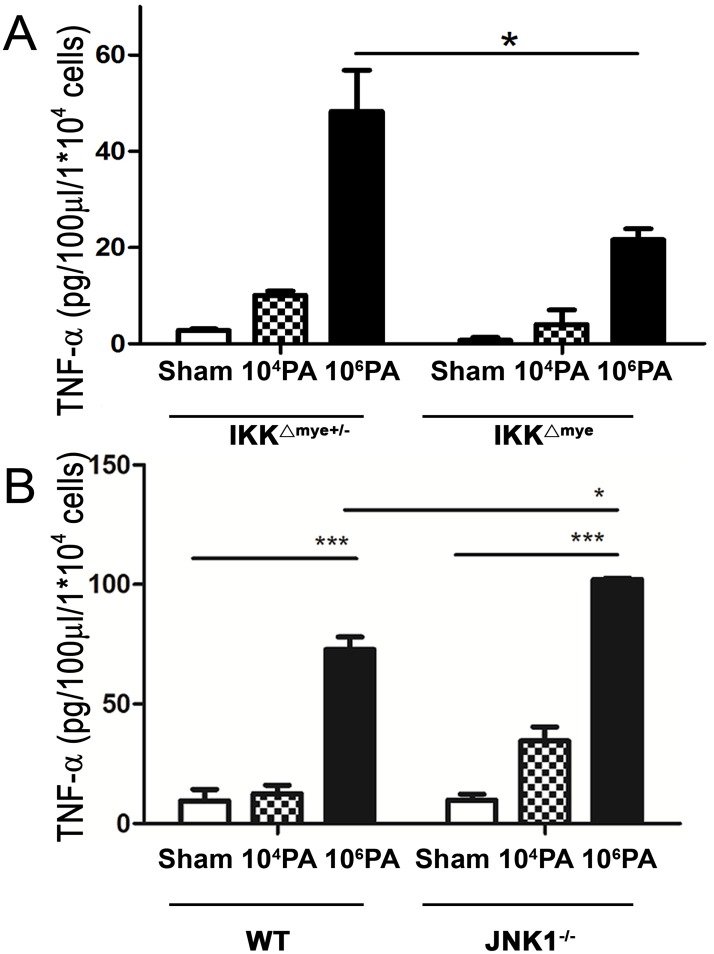
E*x vivo* PA stimulation of AMs from IκB-kinase (IKK)β^ΔMye^ (IKKβ was selectively deleted in macrophages) mice induces less TNF-α production in supernatants than those from JNK1^-/-^ mice as well as WT mice but not IKKβ^ΔMye^ mice. AMs isolated from mice were *ex vivo* stimulated with PA (10^4^ or 10^6^ CFU) for 4 hr and the supernatants were examined. (A) E*x vivo* PA stimulation of AMs from IKKβ^ΔMye^ mice induces less TNF-α production in supernatants than those from IKKβ^ΔMye +/-^ mice. The concentrations of TNF-α were determined by enzyme-linked immunosorbent assay (ELISA). n = 5/group. (B) E*x vivo* PA stimulation of AMs from JNK1^-/-^ as well as WT mice induce TNF-α production in supernatants. n = 5/group. PA, *P*. *aeruginosa*; Super, supernatants from *ex vivo* PA stimulation of AMs. *P<0.05, **P<0.01, ***P<0.001.

To examine the involvement of IKKβ in TNF-α production from PA-stimulated AMs, AMs isolated from IKKβ^ΔMye^ mice were *ex vivo* stimulated with PA (10^4^ or 10^6^ CFU) for 4 h and the supernatants were harvested for assay of the TNF-α levels. The production of TNF-α by AMs from IKKβ^ΔMye^ mice showed a significant 60% decrease as compared to AMs from IKKβ^ΔMye +/-^ mice (control mice) (Fig 7A). This result indicates that PA stimulation induces TNF-α production from AMs mainly through IKKβ dependent signaling pathways.

### Ex vivo PA stimulation induces TNF-α production of AMs from WT and JNK1^-/-^ mice

AMs isolated from JNK1^-/-^ mice were *ex vivo* stimulated with or without PA (10^4^ or 10^6^ CFU) for 4 h and the supernatants were collected. The supernatants were assayed for TNF-α concentrations. E*x vivo* PA stimulation of AMs from WT and JNK1^-/-^ mice induced a significant increase of TNF-α (Fig 7B) in supernatants as compared with the sham group (Fig 7B). The levels of TNF-α were even higher in supernatants collected after 10^6^ PA-stimulated AMs from JNK1-/- mice than those from WT mice. This suggests that TNF-α production by PA-stimulated AMs is through JNK1 independent signaling pathways.

## Discussion

PA is the single-most common pathogen of VAP patients (4/1000 ventilator days) [24]. However, molecular mechanism of PA VAP-induced lung injury remains elusive. In this study, an animal model of PA VAP was established. WT mice instilled with PA (PA colonization) had more neutrophil infiltration in the lungs than instilled with normal saline and the neutrophil infiltration was significantly increased after ventilation with PA colonization. This result suggests that PA colonization significantly enhances MV-induced neutrophil infiltration in the lungs. MV after PA instillation did not increase the bacterial counts in the lungs as compared with PA instillation alone group, suggesting that MV after PA instillation induces lung injury through the enhancement of PA-induced inflammatory process in the lungs rather than the overgrowth of PA. The production of nitrite in BALF can indirectly represent the degree of inflammation [25]. Nitrite concentration in BALF was increased in mice receiving MV after PA instillation. ICAM and VCAM mRNA expression were significantly increased in WT mice receiving MV after PA instillation. Moreover, prior instillation of PA significantly increased the levels of TNF-α, IL-1β and IL-6 in BALF in WT mice after MV. NF-κB is an integrator of different signals involved in inflammatory response [23]. MV did not induced NF-κB DNA binding activity in the lungs. However, MV after PA instillation significantly induced NF-κB DNA binding activity in the lungs. Taken together, these results indicate that PA colonization induces NF-κB DNA-binding activity and greatly enhances MV-induced inflammation and lung injury. Moreover, the enhancement effect of PA colonization on MV-induced ICAM, IL-6, VCAM and MIP-2 mRNA expression in the lungs as well as TNF-α, IL-1β and IL-6 levels in the BALF were all prevented in JNK1^-/-^ mice. Taken together, these results indicate that PA colonization significantly increases PA VAP-induced lung injury and this effect is through the JNK signaling pathway in the lungs.

AMs lie at the first line of lung defense against pathogens. *Ex vivo* alveolar macrophage stimulation assay was used to investigate the involvement of AMs on PA VAP-induced lung injury. AMs release inflammatory mediators into the supernatants after the stimulation with 10^6^ CFU of PA. MV after instillation of supernatants from PA-stimulated AMs induced a significant increase of pulmonary MPO activity as well as total protein concentration in BALF in mice as compared to those receiving ventilation after instillation of supernatants from AMs without PA stimulation. Also, MV after supernatant instillation treatment induced the proinflammatory cytokine (TNF-α and IL-1β) levels in the BALF. *Ex vivo* stimulation of AMs with 10^6^ CFU PA did not induce IL-1β and IL-6 levels in the supernatants (data not shown). However, *Ex vivo* stimulation of AMs significantly increased TNF-α levels in the supernatants. These results indicate that TNF-α production by AMs plays an important role in the stimulatory effect of PA colonization on VAP-induced lung injury. To further evaluate the role of TNF-α, supernatants were replaced by TNF-α protein (100 ng) for instillation. MV after TNF-α instillation even induced a higher lung MPO activity and BALF protein concentration in WT mice than those receiving supernatant instillation and ventilation. Altogether, our results suggest that PA stimulates AMs to produce TNF-α protein in the supernatants. Supernatants from *ex vivo* PA–stimulated AMs could enhance MV-induced lung injury. TNF-α production by AMs plays an important role in MV after PA colonization-induced lung injury. Moreover, AMs isolated from different mice were *ex vivo* stimulated with or without PA (10^4^ or 10^6^ CFU) to examine the different role of IKK and JNK1 signaling pathways in PA stimulation-induced TNF-α production by AMs. E*x vivo* PA stimulation significantly induce TNF-α production by AMs from WT as well as JNK1^-/-^ mice but not from IKKβ^ΔMye^ mice. This result suggests that *ex vivo* PA stimulation of AMs induces TNF-α level in the supernatants mainly through IKK/NF-κB pathways rather than JNK1 signaling pathway. NF-κB DNA binding activity that regulates many important pathological processes of immunity and inflammation was significantly enhanced by MV after PA instillation as compared to PA instillation or ventilation alone group. These results suggest that NF-κB DNA activation-induced TNF-α triggers a cytokine cascade that initiates a series of inflammatory cascades in the lungs.

JNK1^-/-^ mice were used to investigate the role of JNK activation in PA VAP-induced lung injury. There is no significance difference of MPO activity in the lung and total protein in BALF between WT and JNK1-/- KO mice either in control group or PA treatment alone group (data not shown). MV after PA instillation significantly increased expression of IL-6, ICAM, VCAM and MIP-2 mRNA in the lungs and TNF-α, IL-1β and IL-6 in BALF of WT mice but not in JNK1^-/-^ mice. These suggest that PA instillation had no effect on ventilation-induced lung injury in JNK1^-/-^ mice. This indicates that PA colonization induces lung inflammation and enhances ventilation-induced lung injury through JNK signaling pathway in the lungs. MV after instillation of supernatants from ex vivo PA-stimulated AMs did not induce TNF-α levels or total protein concentration in BALF of JNK1^-/-^ mice. MV after TNF-α protein instillation did not induce MPO activity and protein concentration in BALF in JNK1^-/-^ mice. Moreover, e*x vivo* PA significantly induce TNF-α production by AMs from WT as well as JNK1^-/-^ mice. Taken together, these results suggest that PA instillation enhances MV-induced pulmonary inflammation as well as lung injury through JNK signaling pathway in the lungs. JNK1 deficiency does not inhibit the production of TNF-α protein by AMs but significantly decrease PA VAP-induced lung injury through the reduction of inflammation in the lungs. This further corroborates that JNK signaling pathway in the lungs is critical in PA VAP-induced lung injury.

Moreover, mechanical ventilation after supernatant instillation induced a 100% mortality rate at 48 h after ventilation in WT mice. However, mechanical ventilation after supernatant instillation induced less mortality in JNK1^-/-^ mice. PA colonization stimulates AMs to release mediators that along with mechanical ventilation activate AP-1 DNA binding activity in the lungs and induce lung injury. This further corroborates that JNK signaling pathway in the lungs is critical in PA VAP-induced lung injury.

**Fig 8:**
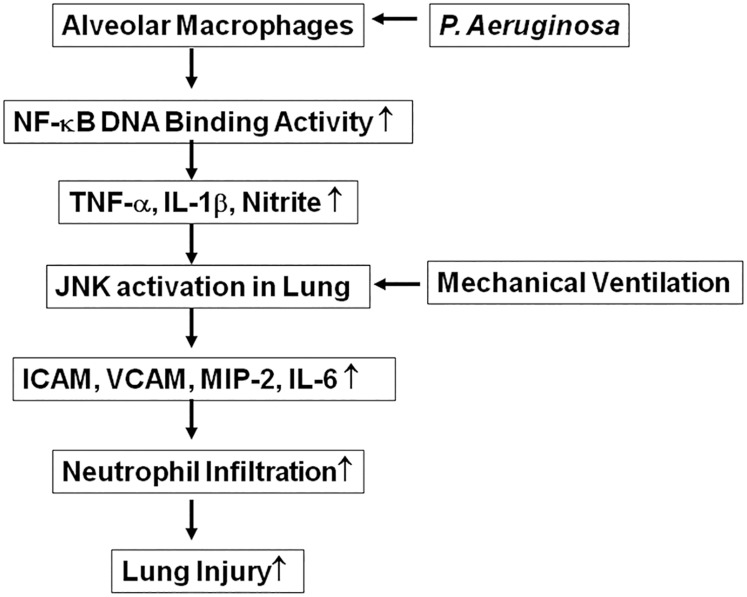
The regulatory mechanism of TNF-α and c-Jun NH2-terminal kinase (JNK) signaling pathways in PA VAP-induced lung injury. *P*. *aeruginosa*, *Pseudomonas aeruginosa*; ICAM, intracellular adhesion molecule; VCAM, vascular cell adhesion molecule; IL, interleukin.

In conclusion, the molecular mechanisms of PA VAP-induced lung injury could be better understood by this study (Fig 8). PA colonization induces TNF-α production of AMs mainly through IKKβ/NF-κB activation. PA colonization enhances the production of TNF-α, IL-1β, and IL-6 in BALF and ICAM as well as VCAM expression in the lungs which induce neutropohil infiltration and lung injury after MV. TNF-α production by AMs induces PA VAP-induced lung injury through JNK signaling pathway in the lungs. The pathogenesis mechanism of PA VAP involves TNF-α production from AMs and JNK signaling pathway in the lungs.

## Supporting Information

S1 Fig Stimulation of AMs with 10^6^ PA had no effect on the production of IL-1β and IL-6 levels in the supernatants. AMs collected from BALF of WT mice were stimulated with live PA for 4 h. After stimulation, supernatants were collected for IL-1β and IL-6 analysis by ELISA. n = 6/group.
(TIF)
