# Supplementary figures and images for: Pseudomonas aeruginosa Ventilator-Associated Pneumonia Induces Lung Injury through TNF-α/c-Jun NH2-Terminal Kinase Pathways
Source: PLoS One. 2017 Jan 6;12(1):e0169267. doi: 10.1371/journal.pone.0169267 (PMC5218563; doi:10.1371/journal.pone.0169267)

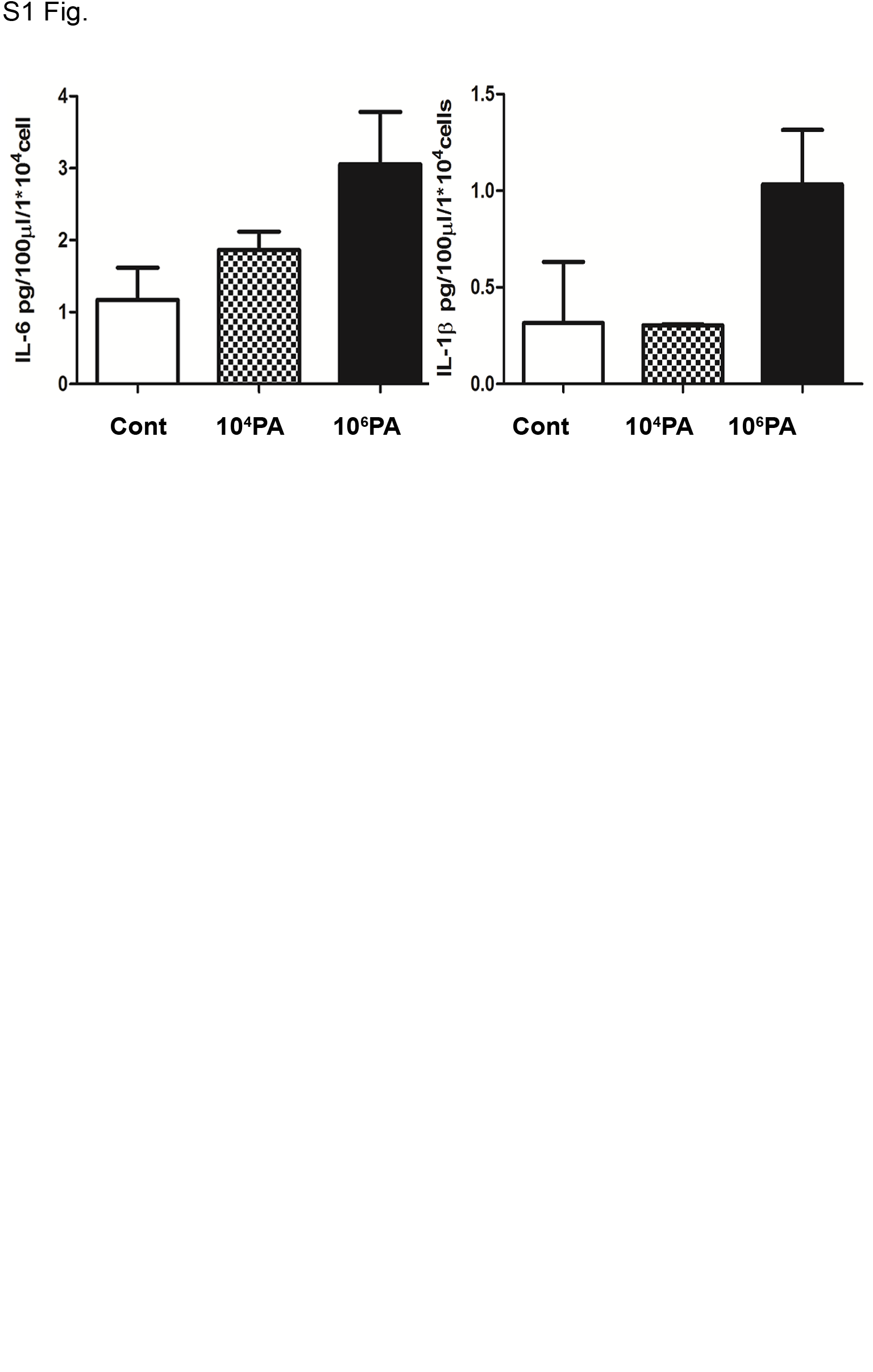

Supplement: S1 Fig — AMs collected from BALF of WT mice were stimulated with live PA for 4 h. After stimulation, supernatants were collected for IL-1β and IL-6 analysis by ELISA. n = 6/group. (TIF) [file pone.0169267.s001.tif]
